# Supplementary material for: Towards guidelines to harmonize textural features in PET: Haralick textural features vary with image noise, but exposure-invariant domains enable comparable PET radiomics
Source: PLoS One. 2020 Mar 16;15(3):e0229560. doi: 10.1371/journal.pone.0229560 (PMC7075630; doi:10.1371/journal.pone.0229560)
Supplement: S1 Fig — Mean activity concentration μc in the VOI as a function of exposure and grouped according to different reconstruction protocols. (a) SR acquisitions, (b) HR and UHR acquisitions. (c) Shown is the absolute range between the lowest and highest values in units of Bel found in first order statistics. Liliefor’s p varied up to 12 orders of magnitude and is not shown to scale. (PDF) [file pone.0229560.s001.pdf]

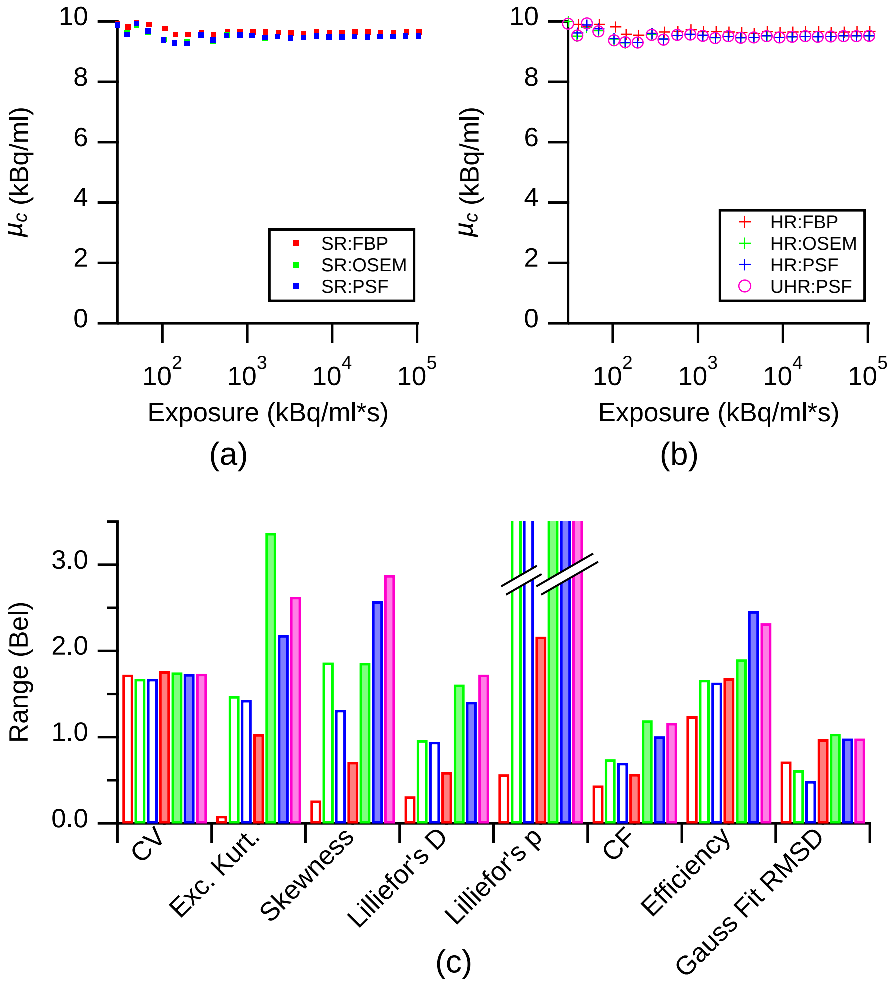

S1 Fig. Variability of first order statistics. Mean activity concentration μ_c in the VOI as a function of exposure and grouped according to different reconstruction protocols. (a) SR acquisitions, (b) HR and UHR acquisitions. (c) Shown is the absolute range between the lowest and highest values in units of Bel found in first order statistics. Liliefor's p varied up to 12 orders of magnitude and is not shown to scale.
