# Supplementary material for: Towards guidelines to harmonize textural features in PET: Haralick textural features vary with image noise, but exposure-invariant domains enable comparable PET radiomics
Source: PLoS One. 2020 Mar 16;15(3):e0229560. doi: 10.1371/journal.pone.0229560 (PMC7075630; doi:10.1371/journal.pone.0229560)
Supplement: S2 Fig — (a) Cumulative histograms of absolute ranges between the lowest and highest values in units of Bel found in all texture features from differently quantized GLCMs. (b) Histograms of differences in feature range from differently quantized GLCMs, i.e. effect size. (PDF) [file pone.0229560.s002.pdf]

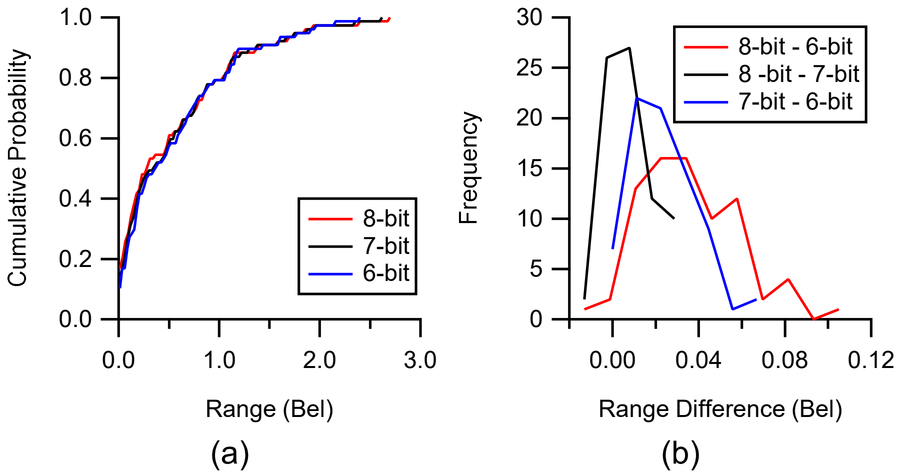

S2 Fig. Effect of GLCM greyscale quantization on magnitudes of textural feature variability. (a) Cumulative histograms of absolute ranges between the lowest and highest values in units of Bel found in all texture features from differently quantized GLCMs. (b) Histograms of differences in feature range from differently quantized GLCMs, *i.e.* effect size.
