# Supplementary material for: Towards guidelines to harmonize textural features in PET: Haralick textural features vary with image noise, but exposure-invariant domains enable comparable PET radiomics
Source: PLoS One. 2020 Mar 16;15(3):e0229560. doi: 10.1371/journal.pone.0229560 (PMC7075630; doi:10.1371/journal.pone.0229560)
Supplement: S3 Fig — Overlaid examples of five full range GLCMs calculated from six differently reconstructed datasets with five selected acquisition durations. GLCM maxima are normalized to unity and are displayed with a logarithmic color scale. Acquisition times are noted adjacent to each SR FBP GLCM in the respective colors. NPairs denotes the number of GLCM pairs analyzed for every color-coded matrix. Color scale in HR:FBP applies to all. Example GLCMs for HR:PSF are not shown, due to their similarity to the UHR:PSF measurements. Greyscales are truncated at 400. (PDF) [file pone.0229560.s003.pdf]

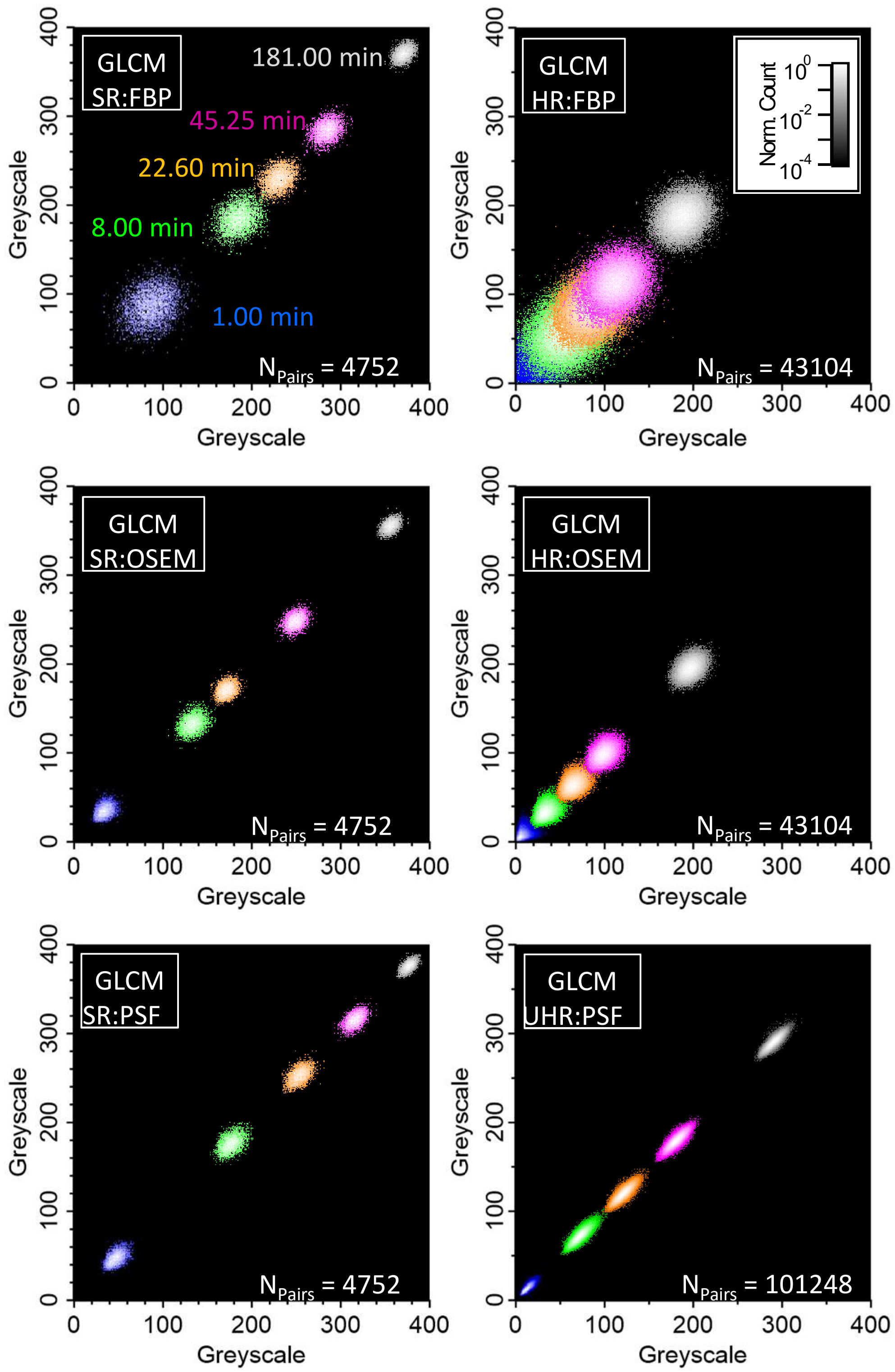

S3 Fig. Overlaid examples of five open range GLCMs Overlaid examples of five full range GLCMs calculated from six differently reconstructed datasets with five selected acquisition durations. GLCM maxima are normalized to unity and are displayed with a logarithmic color scale. Acquisition times are noted adjacent to each SR FBP GLCM in the respective colors. N_{Pairs} denotes the number of GLCM pairs analyzed for every color-coded matrix. Color scale in HR:FBP applies to all. Example GLCMs for HR:PSF are not shown, due to their similarity to the UHR:PSF measurements. Greyscales are truncated at 400.
