# Supplementary material for: Towards guidelines to harmonize textural features in PET: Haralick textural features vary with image noise, but exposure-invariant domains enable comparable PET radiomics
Source: PLoS One. 2020 Mar 16;15(3):e0229560. doi: 10.1371/journal.pone.0229560 (PMC7075630; doi:10.1371/journal.pone.0229560)
Supplement: S4 Fig — Calculating islands of stability for the case of the inverse difference moment (IDM) feature according to the seven different reconstruction protocols, and for full range GLCMs. Plots depict the LOESS curves (colored solid lines) of the data (symbols) with their 99% confidence intervals (dotted lines). Black solid and dotted lines show the respective first derivatives of the Loess curves, with zero crossings at the intersections with the dashed horizontal line. Black crosses mark the islands of stability found for the IDM. The middle legend applies to all but the UHR PSF data. (PDF) [file pone.0229560.s004.pdf]

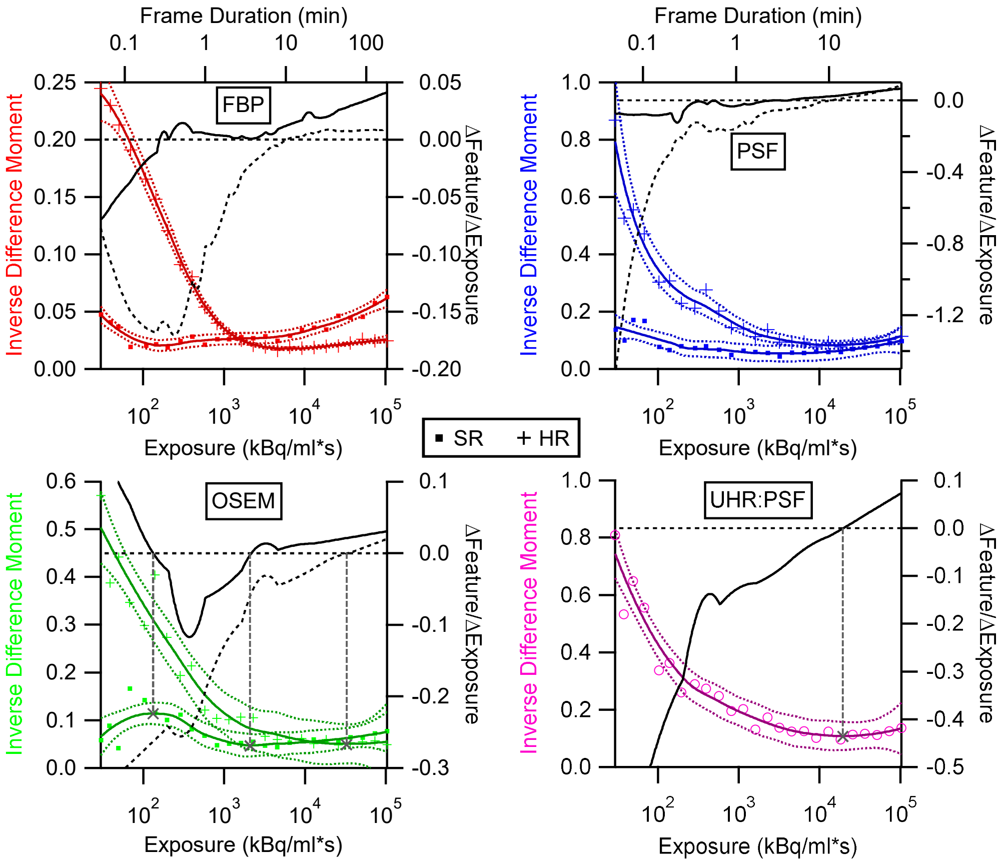

S4 Fig. Islands of stability

Calculating islands of stability exemplified for the Inverse Difference Moment feature according to the seven different reconstruction protocols used and for open range GLCMs. Shown are the LOESS curves (colored solid lines) of the data (symbols) with their 99% confidence interval (dotted lines). Black solid and doted lines show the respective first derivative of the Loess curves with its zero crossing at the intersections with the dashed horizontal line. Black crosses mark island of stability found for the inverse difference moment feature. The middle legend applies to all but the UHR PSF data.
