# Supplementary material for: Towards guidelines to harmonize textural features in PET: Haralick textural features vary with image noise, but exposure-invariant domains enable comparable PET radiomics
Source: PLoS One. 2020 Mar 16;15(3):e0229560. doi: 10.1371/journal.pone.0229560 (PMC7075630; doi:10.1371/journal.pone.0229560)
Supplement: S5 Fig — Exposure dependency of Haralick textural features according to different image resolution and reconstruction algorithm calculated from full range GLCMs with 256 grey levels. Measurement points are shown in the first graph but omitted for clarity in subsequent. Loess curves are shown without associated confidence intervals. (PDF) [file pone.0229560.s005.pdf]

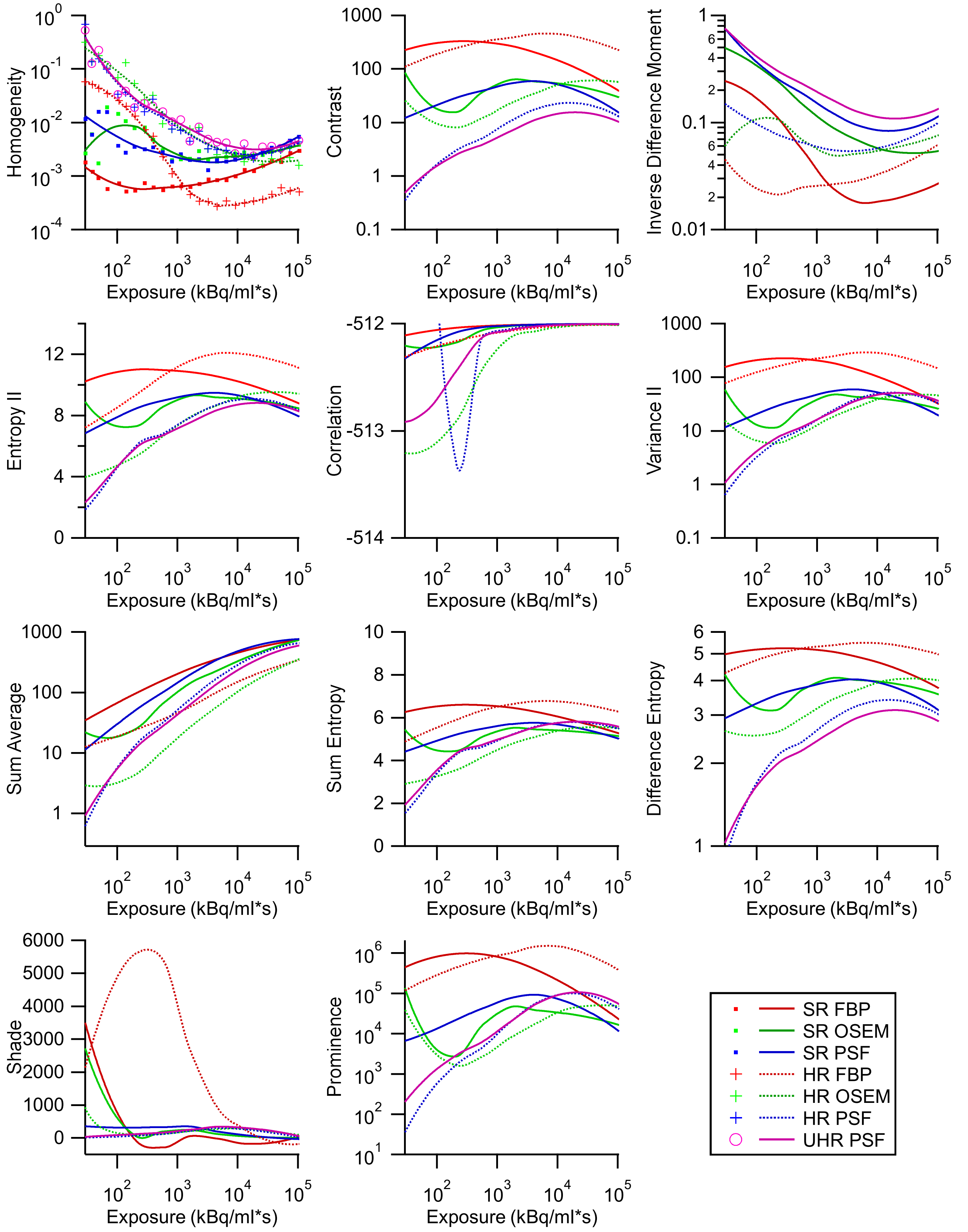

Fig S5. Feature values calculated from full range GLCMs. Exposure dependency of Haralick textural features according to different image resolution and reconstruction algorithm calculated from full range GLCMs with 256 grey levels. Measurement points are shown in the first graph but omitted for clarity in subsequent. Loess curves are shown without associated confidence intervals.
