# Supplementary material for: Towards guidelines to harmonize textural features in PET: Haralick textural features vary with image noise, but exposure-invariant domains enable comparable PET radiomics
Source: PLoS One. 2020 Mar 16;15(3):e0229560. doi: 10.1371/journal.pone.0229560 (PMC7075630; doi:10.1371/journal.pone.0229560)
Supplement: S6 Fig — (a) Variability between the lowest and highest values found in the texture features across all examined exposures calculated form full range GLCMs. (b) Islands of stability in features calculated from full range GLCMs with median exposure as a blue line. *Missing values indicate absence of an island of stability. (PDF) [file pone.0229560.s006.pdf]

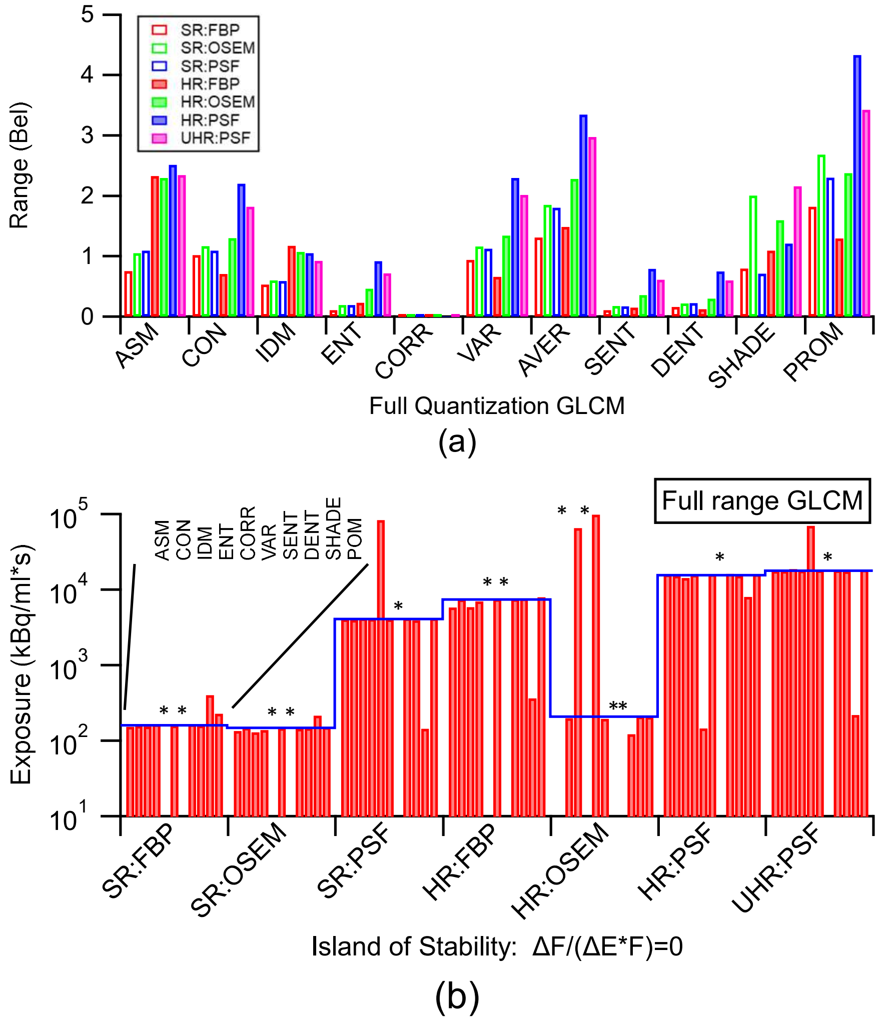

S6 Fig. Variability and islands of stability in features from full range GLCMs. (a) Variability between the lowest and highest values found in the texture features across all examined exposures calculated form full range GLCMs. (b) Islands of stability in features calculated from full range GLCMs with median exposure as a blue line. *Missing values indicate absence of an island of stability.
