# Supplementary material for: Towards guidelines to harmonize textural features in PET: Haralick textural features vary with image noise, but exposure-invariant domains enable comparable PET radiomics
Source: PLoS One. 2020 Mar 16;15(3):e0229560. doi: 10.1371/journal.pone.0229560 (PMC7075630; doi:10.1371/journal.pone.0229560)
Supplement: S7 Fig — Exposure dependency of all examined textural features according to different image resolution and reconstruction algorithm binned into a GLCM with 512 grey levels restricted from zero to a maximal intensity of 25 kBq/ml. Measurement points are shown in the first graph, but omitted subsequently. Loess curves are shown without confidence intervals. (PDF) [file pone.0229560.s007.pdf]

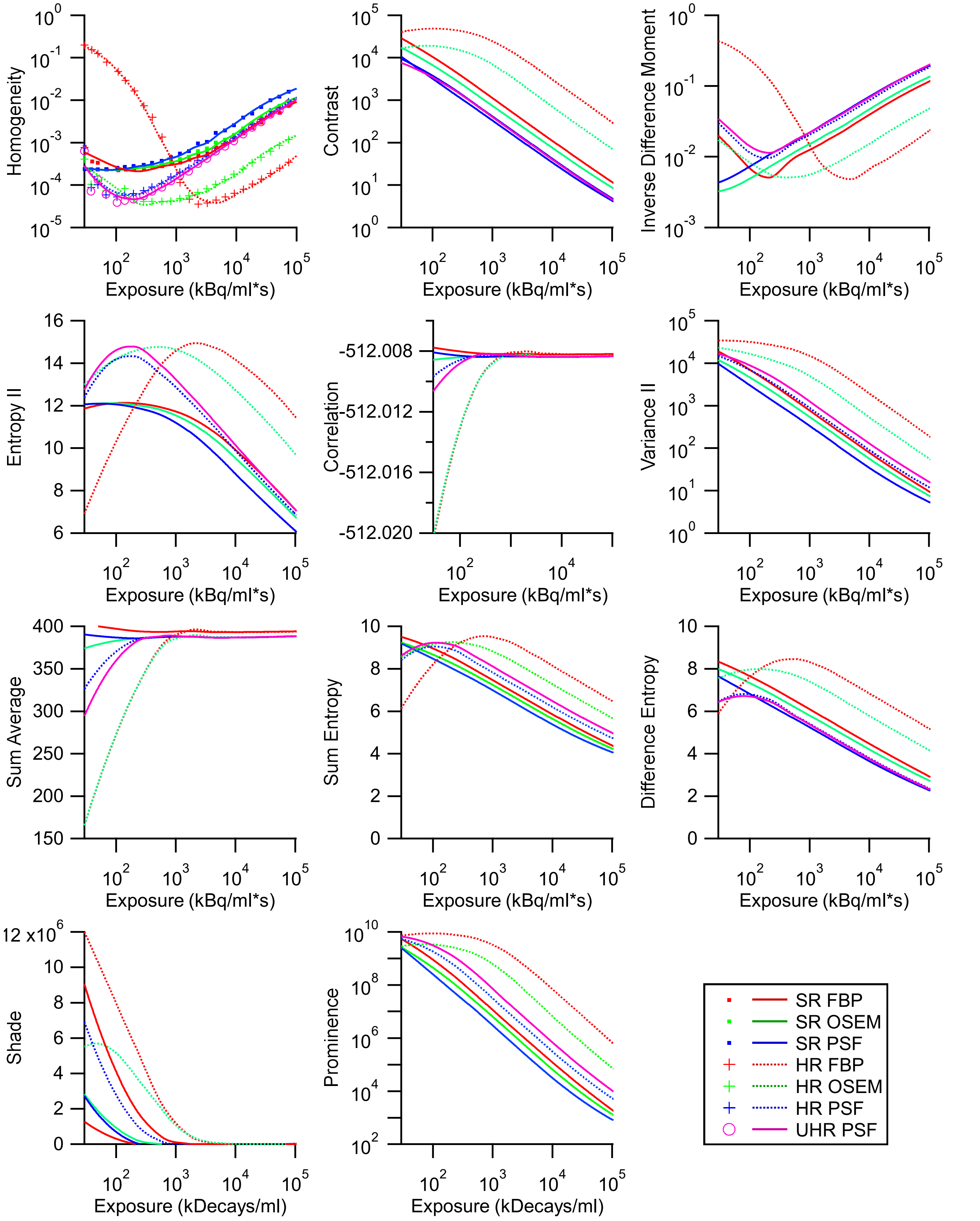

S7 Fig. Effect of restricted GLCM quantizing. Exposure dependency of all examined textural features according to different image resolution and reconstruction algorithm binned into a GLCM with 512 grey levels restricted to a maximal intensity of 25kBq/ml. Measurement points are shown in the first graph but omitted in the following ones, and only the Loess curves are shown. Loess confidence interval are not shown.
