# Supplementary material for: Towards guidelines to harmonize textural features in PET: Haralick textural features vary with image noise, but exposure-invariant domains enable comparable PET radiomics
Source: PLoS One. 2020 Mar 16;15(3):e0229560. doi: 10.1371/journal.pone.0229560 (PMC7075630; doi:10.1371/journal.pone.0229560)
Supplement: S1 Text — Full range and restricted range GLCM quantization. (PDF) [file pone.0229560.s008.pdf]

Towards guidelines to harmonize textural features in PET: Haralick textural features vary with image noise, but exposure-invariant domains enable comparable PET radiomics

George A. Prenosil[1,*], Thilo Weitzel[1], Markus Fürstner[1], Michael Hentschel[1], Thomas Krause[1], Paul Cumming[1,2], Axel Rominger[1], Bernd Klaeser[1,3]

[1]Department of Nuclear Medicine, Inselspital, Bern University Hospital, University of Bern, Bern, Switzerland

[2]School of Psychology and Counselling and IHBI, Queensland University of Technology, Brisbane, Australia

[3]Department of Radiology and Nuclear Medicine, Cantonal Hospital Winterthur, Winterthur, Switzerland

* Corresponding author

E-mail: george.prenosil@insel.ch

Phone: +41 3163-27651

Supporting Material and Methods

To describe effects of GLCM quantization and in contrast to the greyscale mapped GLCMs [6], we designated full range GLCMs, mapping the intensities from zero to the highest intensity found in the entire PET/CT frame onto the 256 GLCM rows and columns. This method resampled the entire quantization range of the PET/CT system to 256 (8-bit) greyscales values and delivers absolute texture values. Furthermore, we designated so-called restricted range GLCMs, with a fixed bin size of 48.8 Bq/ml and 512 bins. These GLCMs were restricted to a maximal intensity of 25 kBq/ml with a lower bound on zero kBq/ml.
