# Supplementary material for: Towards guidelines to harmonize textural features in PET: Haralick textural features vary with image noise, but exposure-invariant domains enable comparable PET radiomics
Source: PLoS One. 2020 Mar 16;15(3):e0229560. doi: 10.1371/journal.pone.0229560 (PMC7075630; doi:10.1371/journal.pone.0229560)
Supplement: S2 Text — Assessing exposure stability of image features when using full range GLCMs. (PDF) [file pone.0229560.s009.pdf]

# Towards guidelines to harmonize textural features in PET: Haralick textural features vary with image noise, but exposure-invariant domains enable comparable PET radiomics

George A. Prenosil[1,*], Thilo Weitzel[1], Markus Fürstner[1], Michael Hentschel[1], Thomas Krause[1], Paul Cumming[1,2], Axel Rominger[1], Bernd Klaeser[1,3]

[1]Department of Nuclear Medicine, Inselspital, Bern University Hospital, University of Bern, Bern, Switzerland

[2]School of Psychology and Counselling and IHBI, Queensland University of Technology, Brisbane, Australia

[3]Department of Radiology and Nuclear Medicine, Cantonal Hospital Winterthur, Winterthur, Switzerland

* Corresponding author

E-mail: george.prenosil@insel.ch

Phone: +41 3163-27651

# Supporting Results

We assessed the effect of using differently quantized GLCMs by looking at the differences in feature value ranges across all Haralick features lumped together, but from GLCMs with different greyscale mapping (S2a Fig). Even though there was a significant reduction in feature value variability upon reducing the number of greyscales (Wilcoxon signed-rank test with Bonferroni correction), the effect size was small (S2b Fig), compared to the effect of varying exposure.

To study the effect of GLCM greyscale mapping, we calculated so-called full range GLCMs with 512 * 512 greyscales, thus mapping the PET data over a range extending from zero kBq/ml to the respective maximum found in the cuboid VOI. S2 Fig shows examples of the full range GLCM dependency on acquisition duration for different reconstructions. Similar to the noise histograms noted above, FBP GLCMs were broader, more Gaussian, and less skewed than were OSEM or PSF GLCMs. The latter two types of GLCM had better alignment on the diagonal, and, with increasing exposure, moved away more quickly from the origin to its upper right corner. Due to their better count statistics, SR datasets also moved faster away from the origin when compared to HR or UHR datasets.

From the full range GLCMs we calculated eleven second-order statistical features and plotted them against exposure (Equations 12 to 22). The resulting scatterplots were smoothed by the LOESS method to obtain continuous curves for every feature. S4 Fig shows LOESS regression curves and the respective 99% confidence intervals obtained thereby for the exemplary case of the IDM feature. With the exceptions of correlation and sum entropy, all features had a non-monotonic behavior with respect to exposure. Therefore, by looking at the zero crossing of the LOESS derivatives, the most stable regions of the texture features were identified where $dF/dE \rightarrow 0$. Zero crossings below exposures of 100 kBq/ml*s were discarded to avoid fringe effects of the LOESS fit. The instable exposures, where $dF/dE \neq 0$, extended over several orders of magnitude (Fig 6) and are to be avoided.

Again taking as an example the IDM feature, S4 Fig shows the LOESS regression curves together with their 99% confidence intervals for the seven different acquisition protocols. While thirteen of the feature-reconstruction combinations lacked so-called islands of stability, some features had more than one zero crossing, for example, the double crossing for the IDM in SR OSEM data (S4 Fig). In these cases, only the earliest crossing was analyzed.

Furthermore, the curves from full range GLCMs (S4 Fig) were less monotonic compared to curves from greyscale mapped GLCMs (Fig 5), varied more (S5a Fig), and had stable maxima or minima (islands of stability) instead of asymptotic plateaus. Exposure values where islands of stability occurred in these curves are shown in S6b Fig, which shows that thirteen reconstruction-feature combinations never reached our stability criterion.
