# Supplementary material for: Towards guidelines to harmonize textural features in PET: Haralick textural features vary with image noise, but exposure-invariant domains enable comparable PET radiomics
Source: PLoS One. 2020 Mar 16;15(3):e0229560. doi: 10.1371/journal.pone.0229560 (PMC7075630; doi:10.1371/journal.pone.0229560)
Supplement: Attachment — Submitted filename: RebuttalLetter.pdf [file pone.0229560.s010.pdf]

INSELSPITAL
UNIVERSITÄTSSPITAL BERN
HÔPITAL UNIVERSITAIRE DE BERNE

UNIVERSITÄT BERN

Universitätsklinik für Nuklearmedizin
Klinikdirektor und Chefarzt
Prof. Dr. med. Axel Rominger

Dr. Mathiew Hatt
Editor, PLoS ONE

Dr. George Prenosil
Department of Nuclear Medicine
Bern University Hospital and University of Bern
Inselspital
3010 Bern, Switzerland
george.prenosil@insel.ch

Bern, 04.10.2019

Rebuttal Letter, Former Manuscript Number: PONE-D-19-18617

Dear Dr. Mathieu Hatt

Following your invitation (case number: 06391075) to resubmit our work with revisions, please find below our rebuttal of the reviewer's comments (red), indicating where we have made changes. Quotes from our new submission are written in italics. For the reviewer's convenience, we marked all lines in the manuscript, which are herein referred to by line number, in yellow. These yellow markups constitute also most of the introduced changes.

Reviewer #1:

The presented manuscript investigates the impact of acquisition time in PET imaging on the stability (value) of texture features. The understanding of uncertainty/noise sources in the transition from qualitative to quantitative analysis is of great importance and often not trivial. It is much easier to study it on phantoms, however with a crucial aim of translating the obtained results into future clinical use. And this point it the largest drawback of this paper. The study was performed on a homogeneous phantom, whereas some custom made heterogeneous phantoms are available in the published literature on radiomics.

The use of a homogenous phantom is completely justified for several reasons, as now stated in the revision:

Line 171 "*to quantify a true texture, one must first be able to identify truly homogeneous areas, where any discerned texture is actually image noise*"

We note that any heterogeneous phantom must be scale-invariant if it is to possess the same features over a range of different resolutions, as used in our work (*c.f.* Lines 137 - 145), and as is required to accommodate multicenter designs. The only readily available scale-invariant phantoms are the cylinders used for daily QA, but custom-made phantoms are impractical for clinical use between centers. Quoting our lines 464 to 467 "*With this work, we present an easily repeatable procedure to identify stable domains, where image noise will not substantially add to textural feature values. These domains should be seen as minimal requirements for comparable radiomics in a multicenter setting.*"

Authors themselves indicated the most important limitations, which prevents from translating these results to clinical studies: depth of the phantom, type of used radionuclide. In addition, the number of studied features is very limited; perhaps other texture matrices are less sensitive to noise (especially the ones calculated on low-pass filtered images).

Lines 480 to 486 in the present submission address the question of VOI depth, and lines 486 to 494 discuss use of different radionuclides.

Indeed, we limit this study to the 11 Haralick features for the very reason their statistical nature makes them prone to noise. Focusing on this single set of features allowed us to make a cause-effect analysis, thus departing from a purely phenomenological study. Because, *de facto,* an infinite number of feature definitions exist, we deemed it more useful to show that attaining feature stability in the face of noise is indeed possible. We also provide a new methodology to assess said stability, rather than simply quoting tabular values that are valid for some particular set of features and our PET/CT settings. As such, our approach is generalizable to other imaging protocols than used in our work (Line 154).

Lines 137 – 142 and lines 499 – 505 address the problem arising from use of low pass filters, which filter noise and texture alike. As explained in detail, the textural features are derived from the highest spatial frequencies of an image. Removing noise at high frequencies by a low pass filter will also remove the textural features under examination and replace them by crosstalk from lower spatial frequencies.

- In general, the manuscript is very long and would benefit from a detailed revision. Since the results itself are quite interesting and the topic is studied in deep, I suggest resubmitting the paper, but instead of putting an emphasize on the influence of the noise on the limited number of texture features, to show texture features as a measure of noise in the image.

We have endeavored to shorten the manuscript, but properly addressing the reviewers` comments necessarily required new text, such that there was little net abridgement (*c.f.* 8255 words in the main text, including references). We believe that the complex issues arising in our study call for adequate explanation and discussion.

Our result that Haralick feature values vary strongly with exposure, but that there are exposure domains with stability, is important, and stand in contrast to previous literature. This seems to us more important than attempting to quantify noise with textural features. In any event, we have incidentally quantified the image noise thoroughly using nine first-order statistics (Fig. 2 and Table 2). In response to the reviews, we have shifted our focus to the key point of exposure invariance, and we follow your suggested title change to read,

“*Towards guidelines to harmonize textural features in PET:*
Haralick textural features vary with image noise, but exposure-invariant
domains enable comparable PET radiomics”.

Detailed comments:

Methods:

- Are the times 3s to 3h really clinically relevant? Please give an example

Indeed, our main concern here is exposure within a VOI, which is the product of acquisition time and activity concentration. Therefore, to arrive at comparable feature values between sites, the required exposures will depend on the sensitivities of the PET/CT systems. These vary over several orders of magnitude depending on the generation of the PET/CT systems. We used three hour acquisition times as limit to obtain the best practicably achievable exposure, thus reflecting approaching a gold standard characterized by the smallest possible noise. Similarly, the three seconds acquisitions represent an according worst case. The extremis make sure, that clinically relevant exposures with strongly varying acquisition times and strongly varying sensitivity of the devices fall inside the examined range.

- Image discretization: 512 gray levels are rarely used in the clinical practice. The higher number of bins causes also higher sensitivity to noise. Small number of bins would smooth the image. In the clinical practice maximum bin number used is around 128, depending on the range of observed intensities.

What was the range in this study? Consider adding different number of bins to your analysis to see what is its influence.

We agree that 9-bit (512 levels) are not relevant to most clinical cases (as far as they exist), and have moved those results to the Supporting Information section. We now focus on a systematic comparison of different bin numbers, comparing feature values calculated from 8-, 7-, and 6-bit GLCMs (Line 206). There has hitherto been no accepted standard for feature definition. We also present results from different binning methods (Fig. 8 and Supporting Results)

- Table 3 – move to supplement

We prefer to leave this table in place, since it would require a lot of text to explain its contents.

- What is the motivation for using FBP? To my understanding this methods is not routinely used.

Indeed, FBP nowadays is rarely used in clinical routine, but we note that Reviewer 2 below argued against using PSF in the present context. Apparently, opinions vary, so we prefer to retain the comparative aspect of our study, also reporting OSEM. The linearity of FBP (with Gaussian post reconstruction filtering) most closely complies with the scale space axioms. Therefore, and assuming the sampling theorem is respected, image structures from FBP are described independently of their scale (within their spatial frequency limits), depending only on the chosen scale (spatial sampling) for the underlying GLCM. This gives more stable and comparable feature values. Furthermore, PSF reconstructions amplify image noise (c.f. Reviewer 2) and use regularization during their iterative image reconstruction. Therefore, we recommend using different reconstructions for image viewing and quantitative analysis (Line 521).

- The so called normalization method is currently a standard in radiomics analysis, either as fixed number of bins or fixed bin size. In both cases, the discretization/normalization is adjusted to the range of intensities observed in the particular patient (vmin corresponds to bin 0). According to the terminology in this manuscript, the analysis on non-normalized images is almost never preformed.

We agree, and have reformulated the paper to focus on greyscale-adjusted GLCMs (Grey level mapping, Lines 220 to 222 and Lines 304 to 306), thus moving most other results into the Supporting Information section. We have carefully adjusted our terminology to avoid ambiguity (*c.f.* Reviewer 2).

Results:

- The recommended scan times are much lower than the ones used in clinical practice (excluding dynamic scans), that would mean that all the clinical scans have satisfactory quality, correct?

This is not quite correct, since (as noted above) the scanning time depends on the sensitivity of the PET/CT system in use *and* the expected uptake. Systems with a tenfold lower sensitivity will thus require ten times longer scans to reach the same domains of feature stability. But, in this case, for example, using Ga-68 instead of Ge-68 might well exceed limitations due to the decay of the phantom, unless the PET/CT were of highest sensitivity. These issues are now mentioned in the revised discussion (lines 458 – 462 and 490 – 491)

- Figure 2 – this is a perfect summary how noise in the image changes with the acquisition time. But the recommendation taken from this figure would be to use rather UHR PSF than a particular scanning time.

We beg to differ; the figure shows the changes in shape and position of GLCMs according to different exposures for explanatory purposes, and no stable exposure values can be derived thereof. Recommendations for exposure values arise from results depicted in the (new) Figures 5, 6 and 7, where the stabilities of particular features are plotted as functions of exposure.

Because the former Figure 2 showed the so-called full range GLCMs, we omit it from the new manuscript. Instead, we use Figures 3 and 4 to show the effect of greyscale mapping, as discussed above.

Discussion

- Page 18, paragraph 2 and 3 – it overlaps to a large extent with introduction, I suggest removing it.

We agreed and deleted half a page of redundant text here.

Reviewer #2:

The authors compare the effect of image exposure on the stability of texture and statistical features using a homogeneous cylindrical phantom containing Ge-68.

1. The authors conclude that exposure generally quite strongly affects feature values, which limits their use for radiomics. However, several aspects of the analysis may have impacted their findings.

I personally would have expected that the mean intensity is more or less stable, but that the variance depends on the exposure. This is more or less shown in figure 4, top left. However, figure 2 and the bottom half of figure 4 show different behaviour. To me it seems that there are two things at play here.

We fear that the figure was misleading, and the reviewer may not fully have understood the different units of the figure: As such, only the relative average in units of greyscale is moving, and the average in units of kBq/ml remains constant. We presented the earlier Figure 4 to explain this effect, by showing the same GLCMs in the two different units. Use of greyscale values is conventional for feature calculation, and thus the resulting feature values are affected by the shift in average values in the GLCM. In the new manuscript, Figures 3 and 4 now show the effect of greyscale mapping as discussed above, which should clarify this point. We added a sentence, that the VOI mean was unaffected by exposure (Lines 263 and 264).

1.1. Firstly, the authors use two different discretization schemes for the GLCM. One is a typical fixed bin number scheme with 256 bins. The other is a bit more uncommon in that it combines a fixed number of bins (512) with a fixed lower bound (0). The latter method is sensitive to the max intensity in the volume, which in term is related to the variance. Lower acquisition duration leads to a wider intensity spread, as shown in figure 1. This may explain the seeming upward shift in figure 2. In reality, the shift is not so much upward with longer acquisition time. Rather the sample error of the mean decreases, which causes the estimated intensity to become better quantified.

We agree entirely. In the earlier paper, we used the first method to show the effect of GLCM quantization. We now place greater focus on a commonly used quantizing scheme, and have moved our report of the full range GLCM results into Supporting Results in the Supporting Information section (S2 to S5 Figs and Lines 377 to 388).

I think it would be much more interesting to perform discretization using another commonly used scheme, namely a fixed bin size scheme with the lower bound on 0. Each bin could represent between 0.10-0.50 kBq/ml. The major advantage of this discretization method is that it is not sensitive to the maximum intensity.

Additional results now presented in Figure 8 use this reviewer`s suggested discretization scheme, which show increased variability and a lack of stable exposure domains. This shows the fixed bin size intensity-discretization scheme to be inferior to the commonly used greyscale mapping, with respect to variability and stability. This comparison gives further support to our recommend use of greyscale mapping (line 523).

1.2. Secondly, since the VOI is in a homogenous region (given sufficiently long measurements), there is not a lot of texture to speak of. However, with the relatively large number of bins in the currently used methods, the authors may induce and measure texture which is not actually present.

This was exactly our intention, to show how texture emerges where there should be none. This observation clearly shows the problems arising from insufficient exposure; noise must be sufficiently low for real texture to emerge. Thus, our approach in imaging a homogenous phantom provides a baseline measurement for subsequent texture detection.

It would probably be better to perform fixed bin number discretization using somewhere between 8 and 64 bins, instead of 256, as this may lead to more stable results. 256 bins is nowadays not a very common choice for that reason.

By reducing the number of bins, information about image texture disappears. Current PET/CT systems use a discretization of >32,000 bins! We are aware of the tradeoff between having sufficient bins for texture detection and increased image noise detection. Therefore, we recommend determining the best exposure for each set of texture definitions before actually deploying them in clinical practice. Nevertheless, as stated above, we have repeated the analysis for 8-, 7-, and 6-bit GLCMs. The new Figures 6, 7 and S1 show the minor effect of varying the number of GLCM greyscales on feature value stability.

1.3. For the same reason I am not sure if PSF-based reconstruction actually makes sense, as the basic assumption of PSF (the measured signal derives from a smaller anatomical region) is easily violated using a phantom with a large homogeneous cylindrical insert. PSF will tend to amplify statistical noise for short acquisition durations.

As noted above, this perspective contradicts Reviewer 1, who objected to FBP. Because opinions and procedures vary, we chose to compare all three commonly available reconstruction algorithms (FBP, OSEM, PSF) in this work. As we state above, determining feature values in homogeneous regions is a prerequisite for detecting any true textures: Texture analysis is bound to fail or give spurious results if homogenous or inhomogeneous regions give spurious texture, actually due to noise. Furthermore, we recommend using different image reconstructions for viewing (PSF or OSEM) and texture analysis (FBP) (line 511).

2. The phantom used by the authors contains a radioactive Ge-68 isotope. Most radiomics studies use a tracer based on the F-18 isotope. The difference in halflife between the two isotopes is large, with 6600 hours for Ge-68 and 1.83 hours for F-18. This means that there may be a large difference in measured activity, and thus counts. If this difference is indeed large, I would doubt the generalisability of the findings. Doubts may exist particularly if F-18 total counts exceed Ge-68 counts by some margin within a similar time frame. Is the total activity in a typical clinical situation for a 3 minute bed time acquired at 75 minutes with an injected dose of 440MBq F-18 tracer comparable to the total activity produced within the same time frame by the Ge-68 isotope?

Indeed, feature results need not be directly comparable between positron emitters. Contrary to the case of Ge-68, F-18 will undergo considerable decay during acquisitions, and does not entail any relevant branching ratio (line 486 - 494). Because acquisition time for capturing features must be adjusted before image acquisition, exposure follows from the integral of (declining) F-18 decays in the regions of interest. In relevant context we

explicitly use the term exposure instead of acquisition time because exposure refers to an actual number of decays and therefore takes into account the decay time AND activity concentration. Experience or literature results constrain the range of tracer uptake likely to be encountered in patients.

To clarify our use of the term "Exposure", which in effect includes decay correction, we added a more detailed definition (lines 95 to 100)

3. The authors' claim that theirs is the first attempt to quantify the effect of exposure time is not correct. I would like to refer to a recent meta-analysis for further information [10.1007/s00259-019-04391-8].

This important meta-analysis appeared around the time of our first submission. We now cite it and discuss its findings. The meta-analysis claims that there is no influence of exposure on the repeatability of feature value analysis. Nevertheless, two original works cited therein show some effect of noise on textural features [10.1016/j.ejmp.2018.05.024] and [10.1371/journal.pone.0159984]. Our present measurements prove a clear effect of count statistics on textural feature values. In elaborating this discussion, we cite four additional literature references in the new manuscript. (Lines 112 to 113, 114 to 116, and lines 495, 498, 501)

4. In figure 1c we have an example that FBP reconstruction may lead to (artificial) negative activity. How does this interact with the full range discretisation scheme that starts at 0 activity?

This happens only for very short exposures, which must be avoided (lines 369 to 371, and line 522): We show that feature stability will arise once negative values cease to appear in FBP images. Furthermore, the revised work now mainly focuses on the greyscale mapped discretization scheme; we prefer to leave aside the problem of negative values in the aforementioned full range discretization scheme.

5. In figure 6 the authors show the order of change over the different exposure levels. I am not sure what the range represents. Is this a log-10 scale so that 0.0 basically means that there is no variation, and 1.0 means a 10-fold change?

Indeed, we use a decadic logarithmic scale, with the units clearly stated as "Bel" directly representing orders of magnitude. The reviewer may be unfamiliar with this denomination of scale.

Does this analysis make sense if features values do not follow a normal distribution, i.e. a non-linear sensitivity to underlying grey level values exists?

Our analysis in Bel units depicts the orders of magnitude in variation to be expected. Exactly because of the nonlinearity of the sensitivity to the grey-level values, we get variability extending over several orders of magnitude. This might be considered a main message of the paper, and such findings cannot be depicted with a linear scale. Furthermore, using a logarithmic scale often converts non-Gaussian distributions into more Gaussian-like distributions.

6. There is likely a spelling error in figure 7a: POM instead of PROM.

We have corrected the error. Former Figure 7b became new Figure 7a. Former Figure 7a became new Figure S5a.

7. I must disagree with the statement by the authors that the comparability of feature values between clinical sites has not been considered. A sizeable assortment of studies has appeared over the years, as may be observed in the meta-analysis referred to earlier.

Indeed, there are previous studies comparing feature values between sites, but hitherto no systematic analysis in terms of exposure. We clarified this point in the Introduction and the Discussion.

8. As remarked by the authors, quantisation indeed affects feature values and may be a source of variability. This is pretty well understood. Again, the authors may refer to the aforementioned meta-analysis and relevant studies included therein.

As noted above we cite and discuss the new meta-analysis (Line 108).

9. The tem “normalised GLCM” as used by the authors may not be clear. I understood this to refer to a fixed bin number discretisation scheme. Grey level co-occurrence matrices themselves are always normalised anyway prior to feature computation, i.e. the sum of all matrix elements is 1. This goes back all the way to the original definitions by Haralick [10.1109/TSMC.1973.4309314]. The co-occurrence matrix-based features as defined by the Image Biomarker Standardisation Initiative follow this convention as well [arxiv.org/abs/1612.07003].

Indeed, there was some ambiguity in the original manuscript. Therefore, we have replaced the term “normalized GLCMs” with “greyscale mapped GLCMs” to distinguish from open range and restricted range GLCMs. Obviously, we normalized all our GLCMs to their number of greyscale-pairs

10. PLOS has an open data policy. The link provided by the authors however leads to a website where access needs to be approved, despite the claim by the authors that “all data are fully available without restriction”.

We have now implemented full public accessibility of the data. We hope that our point-by-point responses dispel any doubts expressed by the reviewers.

Sincerely yours,

Dr. George A. Prenosil, on behalf of the authors
