# Supplementary material for: Towards guidelines to harmonize textural features in PET: Haralick textural features vary with image noise, but exposure-invariant domains enable comparable PET radiomics
Source: PLoS One. 2020 Mar 16;15(3):e0229560. doi: 10.1371/journal.pone.0229560 (PMC7075630; doi:10.1371/journal.pone.0229560)
Supplement: Attachment — Submitted filename: RebuttalLetter2.pdf [file pone.0229560.s011.pdf]

INSELSPITAL
UNIVERSITÄTSSPITAL BERN
HÔPITAL UNIVERSITAIRE DE BERNE

UNIVERSITÄT BERN

Universitätsklinik für Nuklearmedizin
Klinikdirektor und Chefarzt
Prof. Dr. med. Axel Rominger

Dr. Mathiew Hatt
Editor, PLoS ONE

Dr. George Prenosil
Department of Nuclear Medicine
Bern University Hospital and University of Bern
Inselspital
3010 Bern, Switzerland
george.prenosil@insel.ch

Bern, 04.10.2019

Rebuttal Letter, Former Manuscript Number: PONE-D-19-18617

Dear Dr. Mathieu Hatt

Following your invitation (case number: 06391075) to resubmit our work with revisions, please find below our rebuttal of the reviewer's comments (red), indicating where we have made changes. Our previous responses are marked in green. For the reviewer's convenience, we marked all lines in the manuscript, which are herein referred to by line number, with track changes in the PDF manuscript file as done by MS Word.

Journal Requirements:

1. When submitting your revision, we need you to address these additional requirements.

Please ensure that your manuscript meets PLOS ONE's style requirements, including those for file naming. The PLOS ONE style templates can be found at

http://www.journals.plos.org/plosone/s/file?id=wjVg/PLOSOne_formatting_sample_main_body.pdf and http://www.journals.plos.org/plosone/s/file?id=ba62/PLOSOne_formatting_sample_title_authors_affiliations.pdf

We followed the style template

2. We note that you have included the phrase "data not shown" in your manuscript. Unfortunately, this does not meet our data sharing requirements. PLOS does not permit references to inaccessible data. We require that authors provide all relevant data within the paper, Supporting Information files, or in an acceptable, public repository. Please add a citation to support this phrase or upload the data that corresponds with these findings to a stable repository (such as Figshare or Dryad) and provide and URLs, DOIs, or accession numbers that may be used to access these data. Or, if the data are not a core part of the research being presented in your study, we ask that you remove the phrase that refers to these data.

The data previously not shown was placed in the supporting information section in S1 Fig

Additional Editor Comments (if provided):

Both reviewers are in agreement that the revised paper is much improved compared to the original submission. However, the first one is much more reserved, especially with respect to both the overall rationale and the clinical relevance of the work and estimates the most critical points of his first review were not adequatly adressed. The second reviewer has additional comments that will need to be taken into account in the second revision. However, it will require additional results and analyses in order to adequately respond to all initial and new comments of the first reviewer.

Reviewers' comments:

Reviewer's Responses to Questions

Comments to the Author

1. Is the manuscript technically sound, and do the data support the conclusions? The manuscript must describe a technically sound piece of scientific research with data that supports the conclusions. Experiments must have been conducted rigorously, with appropriate controls, replication, and sample sizes. The conclusions must be drawn appropriately based on the data presented.

Reviewer #1: Yes

Reviewer #2: Yes

Feature

2. Has the statistical analysis been performed appropriately and rigorously?

Reviewer #1: Yes

Reviewer #2: Yes

3. Have the authors made all data underlying the findings in their manuscript fully available?

The PLOS Data policy requires authors to make all data underlying the findings described in their manuscript fully available without restriction, with rare exception (please refer to the Data Availability Statement in the manuscript PDF file). The data should be provided as part of the manuscript or its supporting information, or deposited to a public repository. For example, in addition to summary statistics, the data points behind means, medians and variance measures should be available. If there are restrictions on publicly sharing data—e.g. participant privacy or use of data from a third party—those must be specified.

Reviewer #1: No

We added the tag DICOM Files to the file archive (.zip) 20180717 at https://osf.io/36c7s/. Additionally, we uploaded tsv-files (3bit_8bit_Reports.zip) with all the extracted noise and feature values from the DICOM files, analyzed with 3 to 8 bit GLCMS. We added a section "Disclosure", were the availability of the data is mentioned together with the download link (Line 571).

Reviewer #2: Yes

4. Is the manuscript presented in an intelligible fashion and written in standard English?

INSELSPITAL
UNIVERSITÄTSSPITAL BERN
HÔPITAL UNIVERSITAIRE DE BERNE

PLOS ONE does not copyedit accepted manuscripts, so the language in submitted articles must be clear, correct, and unambiguous. Any typographical or grammatical errors should be corrected at revision, so please note any specific errors here.

Reviewer #1: Yes

Reviewer #2: Yes

5. **Review Comments to the Author**

Please use the space provided to explain your answers to the questions above. You may also include additional comments for the author, including concerns about dual publication, research ethics, or publication ethics. (Please upload your review as an attachment if it exceeds 20,000 characters)

Reviewer #1: I would like to thank the authors for their answers to my comments. However, their answers do not provide additional rational for the clinical relevance of this study. Although, the study is interesting in its theoretical merit the conclusions drawn are limited. The studied range of exposures is large an authors failed to address its clinical significance. Additionally, the limited set of studied texture features was not addressed. Perhaps other matrices are less prone to the exposure variation.

Clinical relevance of this study is attested based on two important cited works. One work focused on Haralick features from 6-bit GLCMs measured with a PET/CT similar to our own, and the other study provides time activity curves with exposures in the ranges examined in our work (Lines 427 to 449). To make matters clearer, we have provided easy-to-understand calculations of exposure in this new text. As mentioned in our letter to the editor, we analyzed in-depth a particular set of features, the widely employed Haralick features. Extending this study to include the countless possible feature sets is practically untenable, if we were to apply the same degree of explanatory power. However, we do state at the end of discussion that measurements of other feature classes should be done.

The abstract mentions now the possibility of modern PET/CT systems to attain the stable exposures (Lines 56 and 57).

1. 'The use of a homogenous phantom is completely justified for several reasons, as now stated in the revision: Line 171 “to quantify a true texture, one must first be able to identify truly homogeneous areas, where any discerned texture is actually image noise”

We note that any heterogeneous phantom must be scale-invariant if it is to possess the same features over a range of different resolutions, as used in our work (c.f. Lines 137 - 145), and as is required to accommodate multicenter designs.'

Response: I do not agree that the phantom should be scale-invariant. In real life scenario tracer uptake in a tissue is not scale-invariant and thus the control for voxel size in the analysis is important.

We have to disagree; exactly because of the scale-variance of differently resolved PET/CT acquisitions, the procedure used to measure scale sensitive feature must itself be scale invariant. We have tried to clarify this key issue in lines 505 to 514. But we agree, that image resolution must be adapted to the desired clinical question. This is why we provide results for three commonly used image resolutions.

One should not confuse image resolution with voxel size. The resolution of a PET/CT system is given by its systemic PSF (MTF) convolved with the chosen post-reconstruction filter. The voxel size must just follow accordingly, *i.e.* to the Nyquist-Shannon sampling theorem. For this reason, the problematic of image resolution and texture has been already addressed at lines in the previous submission (Now lines 534 to 540). We extended this paragraph at line 537

2. 'Our result that Haralick feature values vary strongly with exposure, but that there are exposure domains with stability, is important, and stand in contrast to previous literature. This seems to us more important than attempting to quantify noise with textural features. In any event, we have incidentally quantified the image noise thoroughly using nine first-order statistics (Fig. 2 and Table 2). In response to the reviews, we have shifted our focus to the key point of exposure invariance, and we follow your suggested title change to read,'

Response (of the reviewer): here remains the question if the instability is observed for the clinically used exposures, or is the result of wide range used for this study

The newly cited works (Lines 427 to 449) have exposures that clearly encompass the range of exposures used for our analysis. Furthermore, instability over a wide range of exposures and is seen in the slope of the feature value-curves. That's why we are also providing the first deviation in S4 Fig to show this fact. Furthermore, it is important to avoid exposure regimes that lead to unstable features (Line 791 and 792). Our work surely shows the extreme but not the impossible (*c.f.* Lines 427 to 449).

3. 'Indeed, our main concern here is exposure within a VOI, which is the product of acquisition time and activity concentration. Therefore, to arrive at comparable feature values between sites, the required exposures will depend on the sensitivities of the PET/CT systems. These vary over several orders of magnitude depending on the generation of the PET/CT systems. We used three hour acquisition times as limit to obtain the best practicably achievable exposure, thus reflecting approaching a gold standard characterized by the smallest possible noise. Similarly, the three seconds acquisitions represent an according worst case. The extremis make sure, that clinically relevant exposures with strongly varying acquisition times and strongly varying sensitivity of the devices fall inside the examined range. '

Response: I understand this reasoning and the differences in the scanner designs. However, could you give an example of a scanner that allows you for clinical image acquisition within 3s per bed position? My concern is that your range, even though it comprises all the clinically relevant times, is so wide that it actually shows the problem mostly in the range which is not clinically used

It seems that Reviewer #1 has not correctly understood the concept of exposure, as he/she repeatedly refers to acquisition time, which is not germane in this context. On an older PET/CT system with ten-fold lower sensitivity, a 30 s would correspond to our 3 s. On the other hand, with the promise of roughly 14 times better sensitivity, a Siemens Biograph Vision can obtain stable VAR values in about 3.7 min instead of 52 min, as stated in our manuscript. As we have mentioned, acquisitions times have no meaning *per se* if used without the expected activity concentration and the without the characterization of the corresponding PET/CT system. Furthermore, the issue is addressed with the citation and discussion of the two example studies (Lines 427 to 449).

Reviewer #2: The authors provided a revised manuscript that investigates the dependence of grey level co-occurrence matrix features and several statistical features on exposure in a homogenous Ge-68 phantom. In my opinion, the manuscript improved considerably. Particularly the methods and the results section have been made easier to understand by focussing on one type of discretisation scheme.

I have the following comments:

1. The manuscript is quite long, which may discourage readers from reading it completely. Compare, for example, with Radiology, which allows for 3000 words in the main body, JNM (5000 words, incl. references), EJNMMI (6000 words, incl. references). I found the introduction particularly hard to digest. In the introduction the authors raise multiple points that are nonetheless relevant, but make it difficult for the reader to understand what this study is about. I would recommend to shorten the introduction considerably by summarising some of these points, and, if necessary, integrate some into the discussion.

Yes, we agree that this manuscript is wordy, in part due to the complexity of the issues arising in it, and in part due to earlier requests for further elaboration upon review. Nonetheless, we have substantially shorted the introduction by removing the EARL section and some other parts (shown with markups) and by moving other text to the revised “Discussion”. The concept of exposure is now largely presented in the revised “Material and Methods” section, but, for the sake of brevity, is not described in detail in the Introduction. Former Figure 8 was moved to the “Supporting Information” section to become S7 Fig.

2. The results contain subsections named “Grey-level co-occurrence matrices” and “Second order statistical features”. I found this somewhat confusing, as second order statistical features are GLCM features (in this work). I would recommend to change these section headers to be more descriptive, or aggregate them into a single section.

Agreed. Second order features as well as GLCFs are now consistently called Haralick features. Additionally, we have changed the results section from “First order statistical features” to “Exposure dependency of first order statistical features”. The section “grey level co-occurrence matrices” in “Results” has been changed to read “Exposure dependency of grey level co-occurrence matrices”, and the “Results” section “Second order statistical features” is now entitled “Exposure dependency of Haralick features”.

3. I would like to commend the authors for focussing on one resampling method only. However, it would be interesting to see what happens when a lower number of bins is selected. For example, Hatt et al. (10.2967/jnumed.114.144055; figure 2) show that some texture feature values (or their correlation with MATV) do depend on the number of bins. The number of bins chosen by the authors is at the high end of this range, which showed relatively few changes. A question that occurred to me was if features extracted from GLCMs with less columns/rows reach stable values earlier? This could be important to inform guidelines. I would recommend 8, 16, 32 bins in addition to the bin numbers already assessed. These values seem to be used more often, see, e.g. Leijenaar et al. (10.1038/srep11075).

The second resampling method, which was suggested in the earlier review of this manuscript, was retained in the supporting material, as it is brief and potentially helpful future studies based on present results Nevertheless, former “Figure 8” was moved to the “Supporting Information” section to become “S7 Fig”. Following the reviewer’s suggestion, we ran the analysis using 8, 16, and 32 bins (Line 218), and have extended the “Results” section accordingly (Fig. 6 and Fig. 7, Lines 347 to 349), as well as the “Discussion” (Lines 459 and 460). The exercise further documented the relative invariance of Haralick features regarding bin size. Hatt et al. (10.2967/jnumed.114.144055) was introduced at line 349. Leijenaar et al. (10.1038/srep11075) became useful at line 386.

4. Lines 103-105: PET/CT sites may be interpreted as anatomical sites instead of centres, which I think is the intended use.

Yes, we now use the more correct term PET/CT systems (Line 107)

5. Lines 137-139: I am not sure if the reference to nearest neighbourhood operations is necessary. I found it confusing, as it does not seem to bear on the current manuscript.

Yes, this is important, as it is exactly this type of features that arise from neighborhood operations. Other definitions use other operations and might have other exposure dependencies (c.f. outlook). Furthermore, this section was already introduced in response to concerns raised in the previous reviewer. This section was moved to the Discussion (Lines 505 - 514).

6. Lines 142-145 “Additionally, the highest …”: This seems to be more fitting for the discussion.

Yes, c.f. above. Was moved to the discussion.

7. Lines 158-160 “The resulting exposures …”: This seems to be more fitting for the discussion. Moreover, what exposures are clinically relevant?

Yes. We have deleted most of this text, but added some new information specifying the clinical significance in the revised Discussion (Lines 427 to 449). The clinically relevant exposures were added to Fig 5, with data taken from the first example (Ref. [47]).

The abstract mentions now the possibility of modern PET/CT systems to attain the stable exposures (Lines 56 and 57).

8. Lines 171-173 “(to quantify …”. This seems to be more fitting to the introduction.

We guess the reviewer means “Discussion”. Moved to discussion (Lines 491 and 492) to shorten the introduction.

9. Line 202: What is a multi-paradigm software?

We have added a reference explaining said principles of software development (Line 214).

10. Lines 217-219 “The large number …”: I did not understand the justification.

The number of grey levels (information content) of a GLCM must be matched by a sufficiently high number of measurement points (voxels). Otherwise, the GLCM will have void entries were none should exist. A statistical analysis of such GLCMs then becomes unreliable. This caveat is now mentioned in the text (Lines 232 and 233).

6. PLOS authors have the option to publish the peer review history of their article (what does this mean?). If published, this will include your full peer review and any attached files.

If you choose “no”, your identity will remain anonymous but your review may still be made public.

Do you want your identity to be public for this peer review? For information about this choice, including consent withdrawal, please see our Privacy Policy.

Reviewer #1: No

Reviewer #2: No

Sincerely yours,

Dr. George A. Prenosil, on behalf of the authors
